# A double-blind, randomized, multicenter, Italian study of frovatriptan versus almotriptan for the acute treatment of migraine

**DOI:** 10.1007/s10194-011-0325-5

**Published:** 2011-03-25

**Authors:** Marco Bartolini, Maria Adele Giamberardino, Carlo Lisotto, Paolo Martelletti, Davide Moscato, Biagio Panascia, Lidia Savi, Luigi Alberto Pini, Grazia Sances, Patrizia Santoro, Giorgio Zanchin, Stefano Omboni, Michel D. Ferrari, Filippo Brighina, Brigida Fierro

**Affiliations:** 1 Ancona, Italy; 2 G. D’Annunzio University, Chieti, Italy; 3 Ospedale Civile San Vito al Tagliamento, San Vito al Tagliamento, Italy; 4 Sapienza University of Rome, Rome, Italy; 14 IDI, Rome, Italy; 5 Catania, Italy; 6 Department of Neurology, University of Torino, Turin, Italy; 7 Inter Department Headache and Drug abuse Center, Modena University, Modena, Italy; 8 C. Mondino Foundation, Pavia, Italy; 9 Monza, Italy; 10 Department of Neurology, University of Padova, Padova, Italy; 11 Italian Institute of Telemedicine, Varese, Italy; 12 Leiden Centre for Translational Neuroscience, Department of Neurology, Leiden University Medical Centre, Leiden, The Netherlands; 13 Department of Experimental Medicines and Neurological Sciences (BioNec), University of Palermo, Palermo, Italy; 15 Department of Experimental Medicines and Neurological Sciences (BioNec), University of Palermo, Via La Loggia 1, 90100 Palermo, Italy

**Keywords:** Migraine, Frovatriptan, Almotriptan, Patient preference

## Abstract

The objective of this study was to evaluate patients’ satisfaction with acute treatment of migraine with frovatriptan or almotriptan by preference questionnaire. One hundred and thirty three subjects with a history of migraine with or without aura (IHS 2004 criteria), with at least one migraine attack in the preceding 6 months, were enrolled and randomized to frovatriptan 2.5 mg or almotriptan 12.5 mg, treating 1–3 attacks. The study had a multicenter, randomized, double blind, cross-over design, with treatment periods lasting <3 months. At study end patients assigned preference to one of the treatments using a questionnaire with a score from 0 to 5 (primary endpoint). Secondary endpoints were pain free and pain relief episodes at 2 and 4 h, and recurrent and sustained pain free episodes within 48 h. Of the 133 patients (86%, intention-to-treat population) 114 of them expressed a preference for a triptan. The average preference score was not significantly different between frovatriptan (3.1 ± 1.3) and almotriptan (3.4 ± 1.3). The rates of pain free (30% frovatriptan vs. 32% almotriptan) and pain relief (54% vs. 56%) episodes at 2 h did not significantly differ between treatments. This was the case also at 4 h (pain free: 56% vs. 59%; pain relief: 75% vs. 72%). Recurrent episodes were significantly (*P* < 0.05) less frequent under frovatriptan (30% vs. 44%), also for the attacks treated within 30 min. No significant differences were observed in sustained pain free episodes (21% vs. 18%). The tolerability profile was similar between the two drugs. In conclusion, our study suggests that frovatriptan has a similar efficacy of almotriptan in the short-term, while some advantages are observed during long-term treatment.

## Introduction

Millions of people worldwide suffer from migraine, a chronic, recurrent, disabling, neurovascular disorder [1]. Triptans are selective serotonin 5-HT1B/1D receptor agonists, generally considered as safe and effective acute migraine drugs [2, 3]. Since the introduction of the first triptan, sumatriptan, almost 20 years ago, several triptans with different pharmacokinetic and pharmacodynamic properties, and thus different efficacy and safety profiles, have been developed [3, 4]. Some of them are labeled as fast triptans and share similar characteristics with sumatriptan, the prototype triptan, displaying a rapid dose-dependent efficacy, with a higher risk of adverse effects, while others seem to have a more delayed onset of the relieving effect on migraine symptoms, but a more prolonged duration of action and a reduced rate of recurrent migraine attacks [5].

Frovatriptan is one of the most recent triptans, developed in order to provide a drug with a clinical potential for a long duration of action and a low likelihood of side effects and drug interactions [6–8]. Randomized clinical studies and post-marketing surveys showed significantly improved effectiveness and tolerability ratings with frovatriptan as compared to previous acute therapies, including triptans, analgesics and non-steroidal anti-inflammatory drugs [9–12].

In the recent past, three double-blind, randomized, head-to-head trials have compared safety of frovatriptan with that of sumatriptan [13], and efficacy and safety of frovatriptan with that of rizatriptan [14] and zolmitriptan [15]. In order to add new information to existing ones, a cross-over study was setup to directly compare in the same subjects efficacy and tolerability of frovatriptan versus almotriptan. The latter is a relatively fast acting triptan, which showed in several randomized studies, lack of clinically relevant pharmacokinetic interaction with other drugs, adverse reactions rate similar to placebo, and good anti-migraine efficacy as compared to other triptans [16, 17].

The study had been designed to assess efficacy by analyzing traditional migraine treatment endpoints and also by considering patient’s preference to treatment [18].

## Methods

### Study population

The study included subjects of male or female gender, 18–65 years old, with a current history of migraine with or without aura, according to International Headache Society (IHS) 2004 criteria, and with at least one, but no more than 6 migraine attacks per month for 6 months prior to entering the study [19].

Patient could not be enrolled in the study in case of: (a) uncontrolled hypertension; (b) ischemic heart disease; (c) cardiac arrhythmias or symptomatic Wolff-Parkinson-White syndrome; (d) previous stroke or transient ischemic attack; (e) severe liver or renal impairment; (f) any other severe or disabling medical condition; (g) history of alcohol or analgesic or psychotropic drug abuse; (h) known hypersensitivity to study drugs; (i) previously demonstrated inadequate response to at least two triptans; (j) current use of propranolol or ergothamine (and its derivatives) as a prophylactic agent; (k) current use or use in the previous 2 weeks of MAO-inhibitors; (l) use of either test medication to treat any one of the last three episodes of migraine; (m) other headaches that have been lasting for more than 6 days. Pregnant women and breast-feeding mothers were excluded as well, while women with childbearing potential but not practicing an effective method of birth control were to be submitted to a pregnancy test, if clinically indicated.

Written informed consent was obtained from all patients prior to their inclusion into the study. The study was approved by the Independent Institutional Review Boards of the study centers.

### Study design

This was a multicenter, randomized, double blind, cross-over study, including 12 centers across Italy (see Appendix). Each patient received frovatriptan 2.5 mg or almotriptan 12.5 mg in a randomized sequence: after treating 3 episodes of migraine in no more than 3 months with the first treatment, the patient had to switch to the other treatment. After treating 3 episodes of migraine in no more than 3 months with the second treatment, each patient was asked to assign preference to one of the treatments according to a questionnaire with a preference score graded from 0 to 5.

The study involved three visits and each patient’s participation time in the study had not to exceed 6 months from randomization. Subjects having no migraine episodes during one of the two observation periods were excluded from the study.

During the randomization visit, after signing written informed consent, subjects provided a medical treatment and migraine history. A physical and neurological examination and pregnancy test (if appropriate) were performed. Blood pressure and heart rate were measured for all subjects. The degree of migraine-associated disability (MIDAS questionnaire) was also completed. At the end of the visit a headache diary documenting characteristics of headache pain and associated symptoms was dispensed with study medication. Subjects were instructed to treat at least 3 migraine episodes occurring in no more than 3 months and to come for the second visit. On this occasion use of concomitant medications and occurrence of adverse events (from diary) were checked, blood pressure and heart rate were recorded, and a pregnancy test performed, if deemed necessary. The same procedures were carried out at the end of the second study treatment period or at the early withdrawal visit, together with the administration of the patient’s preference questionnaire.

Patients were instructed to take one dose of study medication as early as possible after the onset of migraine attack. If insufficient relief had been obtained after 2 h, patients were allowed to take a second dose of study medication, with a maximum daily intake of two doses. In case of insufficient relief 1 h after the intake of the second dose of the study medication, patients were allowed to take a rescue medication. Alternate rescue medication could not include triptans, or contain ergotamine or its derivatives, or propranolol.

### Data analysis

The primary study endpoint was the between-treatment comparison of the direction and average strength of preference at the end of the study, measured on a scale from 0 (no preference) to 5 (strong preference) [14, 15]. The rate of patients expressing a preference and reason for preference were also calculated.

The hypothesis was that a superiority of one treatment against the other had to occur in the presence of a difference of +1.0 with a standard deviation of 2.375. Considering a two-tailed test with a 0.05 significance level and an 80.7% power, the estimated number of patients to be randomized was 120 (including a 25% drop-outs), 60 for each treatment group.

The primary analysis population was the intention-to-treat population, formed by all patients randomized to any of the two treatment sequences, having not positively refused to receive either study treatment, having treated at least one episode of migraine with both medications and who expressed their preference at study termination. The per-protocol population, i.e., patients of the intention-to-treat population displaying no protocol violations, was used for confirmatory analysis.

Secondary study endpoints were quantified according to IHS Guidelines [19]. In summary, these endpoints were: (a) the number of pain free episodes at 2 h (absence of migraine episodes at 2 h after intake of one dose of study drug ± rescue medication), (b) recurrence, assessed as stated in the protocol, i.e., an episode of migraine occurring within 48 h from the previous one, after a period without migraine, and also more appropriately as an episode which is pain free at 2 h and headache of any severity returns within 48 h; (c) the number of sustained pain free episodes within 48 h (migraine attack which is pain free at 2 h, does not recur and does not require the use of rescue medication within 48 h), (d) and the number of pain relief episodes at 2 h (defined as a decrease in migraine intensity from severe or moderate to mild or none). Evaluation of the rate of pain free episodes, recurrence and sustained pain free episodes was also done for the subgroup of subjects with migraine attacks treated no more than 30 min from their onset (early intake).

Tolerability analysis was applied to all randomized patients, by calculating the incidence of adverse events during the treated attacks and changes in vital signs during the study.

Continuous variables were summarized by computing average values and standard deviation (SD), while categorical variables by computing the absolute value and the frequency (as percentage). Preference scores were compared between treatment groups by analysis of variance. Secondary endpoints were compared between groups by generalized estimating equation analysis. Kaplan–Meyer curves for cumulative hazard of recurrence over the 48 h were also drawn. *P* value refers to the statistical significance of between-treatment difference. The level of statistical significance was kept at 0.05 throughout the whole study.

## Results

### Baseline demographic and clinical data

**Fig. 1:**
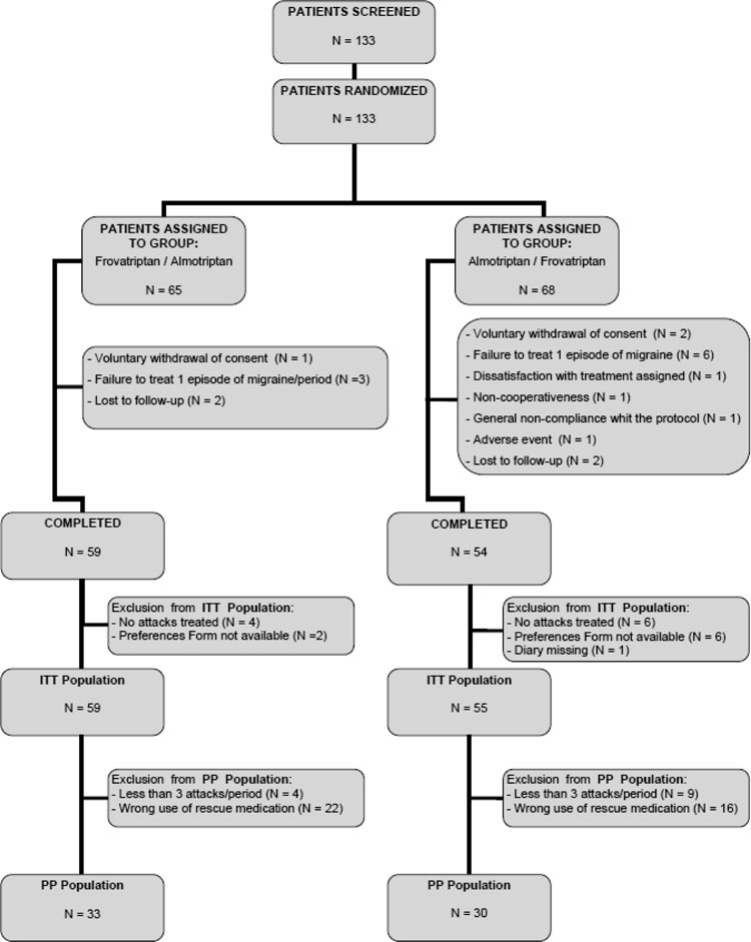
Flow diagram of participants throughout the study

The flow diagram of participants throughout the study is shown in Fig. 1. Overall, 133 patients were screened and randomized to active treatment. Of these patients, 113 completed the study and 20 prematurely withdrew from the study because of failure to treat one episode of migraine (*n* = 9), lost to follow-up (*n* = 4), withdrawal of consent (*n* = 3), dissatisfaction to assigned treatment (*n* = 1), occurrence of an adverse event (*n* = 1), protocol violation (n = 1), lack of cooperation (*n* = 1).

The intention-to-treat population consisted of 114 patients, while the per-protocol population included 63 patients: 51 patients were considered major protocol violation (13 patients treated less than 3 attacks and 38 made wrong use of rescue medication) (Fig. 1). Table 1 shows main demographic and clinical characteristics of the patients of the intention-to-treat population at the time of randomization. Most of the subjects enrolled (84%) were of a female gender, this explaining the high rate of premenstrual attacks (70% of patients treated at least one premenstrual attack). The vast majority of attacks were of moderate or severe intensity (97% of patients had at least one moderate or severe attack). Baseline data for the per-protocol population did not differ from those of the intention-to-treat population (data not shown).

**Table 1:** Demographic and clinical data of the 114 patients of the intention-to-treat population at the time of randomization

	*n* = 114
Age (years, means ± SD)	40 ± 10
Females (*n*, %)	96 (84)
Height (cm, means ± SD)	165 ± 6
Weight (kg, means ± SD)	65 ± 12
Age at onset of migraine (years, means ± SD)	18 ± 8
Migraine attack duration >2 days (*n*, %)	29 (25)
MIDAS score (means ± SD)	23 ± 16
No use of triptans in the previous 3 months (*n*, %)	93 (82)
Premenstrual attacks (*n*, %)^a^	155 (28)
Patients with at least one premenstrual attack (*n*, %)	67 (70)
Migraine with aura (*n*, %)^b^	64 (10)
Patients with at least one attack with aura (*n*, %)	24 (21)
Moderate or severe attacks (*n*, %)^b^	532 (80)
Patients with at least one moderate or severe attack (*n*, %)	111 (97)
Study drug intake <30 min (*n*, %)^(2)^	255 (38)
Patients with at least one study drug intake <30 min (*n*, %)	83 (73)

Data are shown as mean (±SD), or absolute (*n*) and relative frequency (%)

^a^Numbers refer to number and frequency of attacks as respect to overall number of attacks in the female patients

^b^Numbers refer to number and frequency of attacks as respect to overall number of attacks

### Primary end-point

In the 114 subjects of the intention-to-treat population the preference score averaged to 3.1 ± 1.3 for frovatriptan and to 3.4 ± 1.3 for almotriptan (*P* = NS); 63% of patients expressed a clear preference for a triptan (29% for frovatriptan and 34% for almotriptan, *P* = NS). The proportion of patients with a moderate to very good grade of overall satisfaction was slightly, but not significantly, larger under frovatriptan (75% vs. 70% almotriptan).

The most common reasons for preferring one triptan were the rapid action (51.4% frovatriptan and 55.0% almotriptan), prevention of aggravation (13.5% and 2.5%), and reduction of severity (13.5% and 15.0%): no significant differences were observed between treatments for these data. Additional preference results will be published in details elsewhere. Results of per-protocol analysis for the primary end-point were superimposable to those of the intention-to-treat population (data not shown).

### Secondary end-points

Results of the analysis of secondary end-points for the intention-to-treat population are summarized in Table 2. At 2 h, the rates of pain free (30% with frovatriptan vs. 32% with almotriptan) and pain relief episodes (54% with frovatriptan vs. 56% with almotriptan) were not significantly (*P* = NS) different between the two treatments. Sustained pain free episodes were also similar between the two groups (21% frovatriptan vs. 18% almotriptan; *P* = NS).

**Table 2:** Results for the secondary study endpoints

	Frovatriptan	Almotriptan	*P*
Pain free episodes at 2 h	99 (30)	104 (32)	NS
Recurrent episodes (protocol definition)	93 (28)	115 (34)	<0.05
Recurrent episodes (IHS definition)	30 (30)	46 (44)	<0.05
Pain relief episodes at 2 h	143 (54)	144 (56)	NS
Sustained pain free episodes	69 (21)	58 (18)	NS

Data are shown for the intention-to-treat population and reported as absolute (*n*) and relative (%) frequency. *P* refers to the statistical significance of the difference between the two treatment groups

**Fig. 2:**
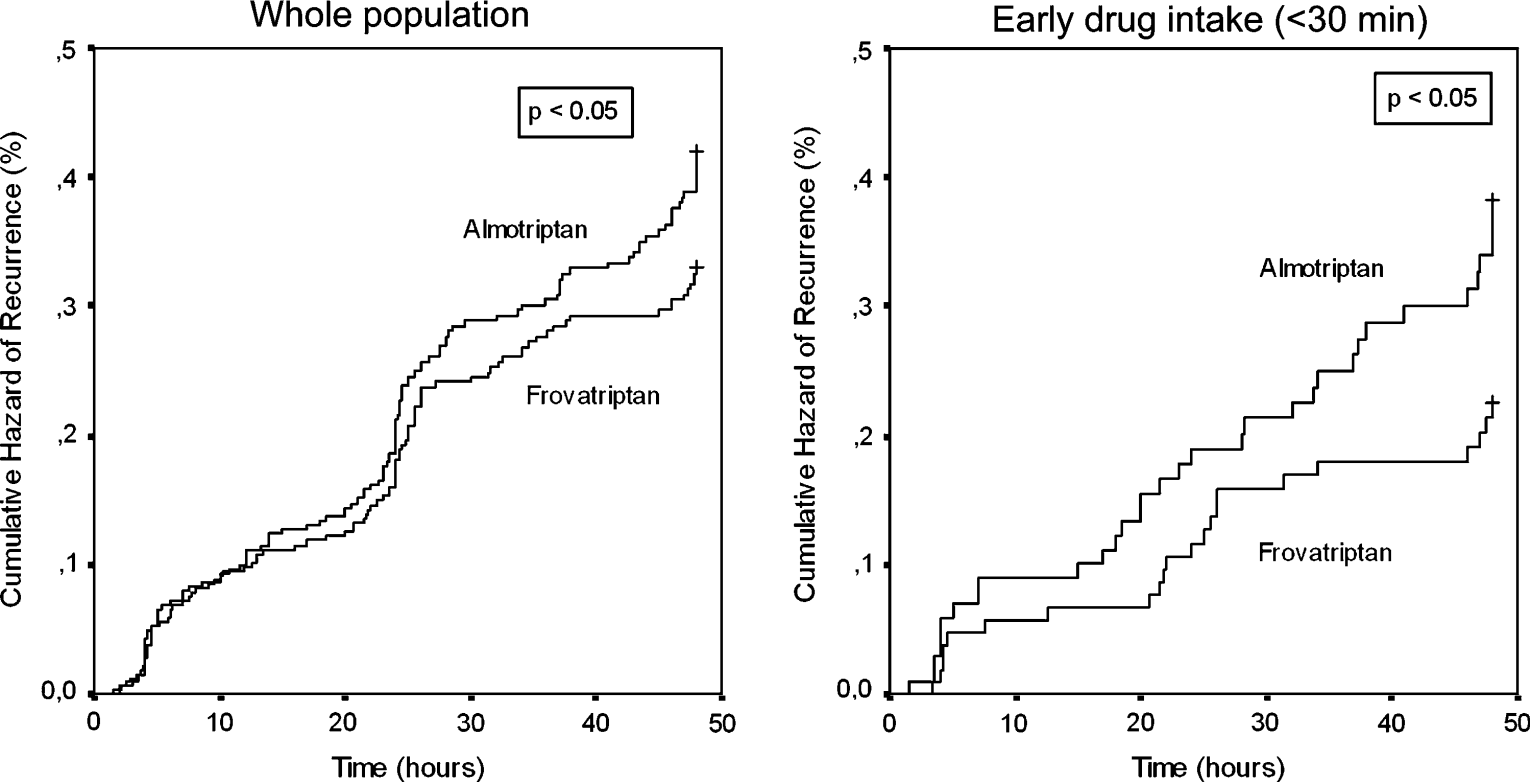
Cumulative hazard of recurrence over the 48 h during treatment with frovatriptan or almotriptan, in the 114 patients of the whole study population and for attacks for which the drug was taken within 30 min from the onset of the episode

Conversely, the rate of recurrent episodes at 48 h was significantly (*P* < 0.05) lower under frovatriptan, both when defined according to the protocol (28% vs. 34% almotriptan) or to IHS criteria (30% vs. 44%). This was the case also when recurrence was expressed as a cumulative hazard ratio over the observation period (Fig. 2, left panel).

A superiority of frovatriptan over almotriptan with regards to headache recurrence was also observed for the 255 (38%) attacks (83 patients, 73% of patients of the intention-to-treat population) for which the drug was taken within 30 min from the onset of the episode (Table 3; Fig. 2, right panel). In this subgroup of the main study population rate of pain free episodes at 2 h and of sustained pain free episodes, did not significantly differ between the two treatments (Table 3).

**Table 3:** Results for the secondary study endpoints in the subgroup of 83 patients with early study drug intake (<30 min)

	Frovatriptan	Almotriptan	*P*
Pain free episodes at 2 h	48 (35)	42 (32)	NS
Recurrent episodes (protocol)	22 (20)	33 (32)	<0.05
Recurrent episodes (IHS definition)	16 (21)	29 (38)	<0.05
Sustained pain free episodes	32 (27)	23 (21)	NS

Data are shown for the intention-to-treat population and reported as absolute (*n*) and relative (%) frequency. *P* refers to the statistical significance of the difference between the two treatment groups

Analysis of the per-protocol population for the secondary end-points gave results similar to those of the intention-to-treat population (data not shown).

### Tolerability

Analysis of tolerability was carried out in 123 patients.

Adverse events were reported by 13 patients during treatment with frovatriptan and by 12 patients during treatment with almotriptan (10.6% vs. 9.8% of treated patients) for an overall number of 52 adverse events (28 under frovatriptan and 24 under almotriptan). Most of the events were of a mild or moderate intensity (89%), and no serious adverse events were recorded during the study. No patients under frovatriptan prematurely withdrew from the study while 1 patient under almotriptan did.

Side effects attributed to study treatment were 31 (60% of total events) and occurred in 4 patients during frovatriptan and in 6 patients during almotriptan treatment (*P* = NS). As reported in Table 4 the number of drug-related adverse events was not significantly different between the two groups, though a larger prevalence of angina-like symptoms (palpitation or tachycardia, thoracic constriction or tightness) was observed under almotriptan (7 vs. 1 under frovatriptan).

**Table 4:** Distribution of absolute numbers of drug-related adverse events between the two treatment groups, in the 123 patients of the safety population

	Frovatriptan			Almotriptan			All
	Intensity			Intensity			
	Mild	Moderate	Severe	Mild	Moderate	Severe	
Asthenia	–	2	–	–	–	–	2
Nausea or vomiting	–	2	–	–	3	–	5
Palpitation or tachycardia	1	–	–	2	–	1	4
Thoracic constriction or tightness	–	–	–	–	2	2	4
Sensation of being dazed	–	–	1	–	–	–	1
Dry or sticky mouth	4	–	–	–	3	–	7
Abdominal pain or diarrhea	–	–	–	–	1	1	2
Anxiety	–	–	–	1	1	–	2
Other	–	2	1	1	–	–	4
Total adverse events	13			18			31
Total patients (%)	4 (3.3)			6 (4.9)			10 (8.2)

Treatment was accompanied by no relevant changes in vital signs, ECG or results of cardiovascular examination.

## Discussion

Acute treatment of migraine with frovatriptan and almotriptan resulted in similar proportions of pain relief and pain free episodes at 2 h, indicating a superimposable short-term efficacy between the two triptans. However, frovatriptan had a more sustained relieving effect on migraine symptoms, with lower rates of headache recurrence over the 48 h than almotriptan. Interestingly, results observed in the subgroup of subjects treating attacks within 30 min after their occurrence was similar to those of the main study population. These differences in efficacy between the two study drugs may be largely attributable to the different pharmacokinetics of the two drugs. Indeed, almotriptan has a slightly shorter time to maximum concentration than frovatriptan [5, 6], this possibly explaining why major benefits in migraine treatment are usually reported under almotriptan when the drug is administered within an hour of migraine onset and particularly when pain is mild [16, 17]. Frovatriptan has a longer half-life than almotriptan (25–26 h vs. 3–4 h), and has one of the greatest 5-HT_1B_ binding receptor affinity among triptans and multiple pathways metabolism, which might explain why frovatriptan, unlike almotriptan, greatly reduced the risk of migraine recurrence [20].

Patient’s preference for one drug or the other did not differ between the study treatments. Frovatriptan was chosen mainly because of the rapid onset of action (54.1% of patients), reduction in pain severity (13.5%), and prevention of aggravation (13.5%). The fact that more than half of the patients preferred frovatriptan for its rapid activity and that similar short-term efficacy was observed between the two groups of patients, also in the subgroup with early drug intake, confirms what was recently reported in a long-term open label study, namely that frovatriptan may provide a remarkably fast and high headache response in more than one-third of migraineurs, labeled as “rapid responders” [21].

Previous direct comparisons between frovatriptan and almotriptan are lacking. However, our results are in line with published indirect comparisons, based on placebo controlled studies [22, 23]. In a review of five randomized, double-blind, placebo-controlled studies, headache response rate at 2 h (pain relief) for frovatriptan ranged between 38% and 40% before placebo correction, while it was slightly higher in our study (54%) [22]. In six studies, pain relief at 2 h under almotriptan averaged to 60–61% before placebo correction (56% in our study) [9, 22–24].

In previous studies, frovatriptan was more effective than placebo in rendering patients pain free at 2 h (12% of patients) [6]. In two different meta-analyses, rate of pain free at 2 h with almotriptan was 35–36%, a proportion very similar to that of our study (31%) [22, 23]. The additional finding of our study is that proportion of pain free episodes at 2 h (30%) under frovatriptan was much higher than that observed in the abovementioned placebo-controlled studies, but it was similar to that found in two recently published studies with the same design and a similar sample size [14, 15]. In such studies rate of pain free ranged between 26% and 33% with frovatriptan, a frequency similar to that of the two comparators (rizatriptan 39% and zolmitriptan 31%).

Headache recurrence at 24 h averaged to 17% with frovatriptan [9] and to 25–27% with almotriptan in former meta-analyses [9, 22]: in our study headache recurrence was assessed more properly within the 48 h, as indicated by IHS guidelines [19], and was significantly less frequent under frovatriptan than almotriptan (30% vs. 44%). This is in line with results of open label naturalistic studies and meta-analyses of placebo-controlled efficacy studies, which suggest that in a real world setting frovatriptan is associated with a lower rate of migraine recurrence than with other triptans [25]. Post-marketing surveys also indicate that the long duration of action of frovatriptan appears to confer other benefits such as greater patient satisfaction, with over 80–90% of patients and physicians rating frovatriptan therapy as very good or good, both in terms of efficacy and tolerability [10–12, 25].

The good long-term efficacy of frovatriptan shown in our study seems to support indication of frovatriptan for those patients requiring a prolonged duration of action, with a sustained effect and less side effects. Pharmacokinetic features of frovatriptan may make it suitable for patients who need prophylaxis such as in menstrual-related migraine and in patients with long-duration or recurrent migraine attacks [24, 26, 27]. In these patients early use of frovatriptan has been shown to be associated with a greater benefit [12, 28]. Conversely, almotriptan may be useful in those patients requiring a rapid pain relief [16, 17].

In terms of tolerability, no significant difference in the prevalence of adverse drug reactions was observed between the two study treatments, although a trend to a higher prevalence of angina-like symptoms was observed under almotriptan. The similar tolerability profile between the two drugs might be regarded as a positive feature in favor of frovatriptan, since it is well known that almotriptan is one of the best tolerated triptans, showing a placebo-like tolerability profile [16, 21].

In conclusion, our multicenter, randomized, double-blind, head-to-head study suggests that frovatriptan and almotriptan are similarly preferred in patients with acute migraine attacks and have a similar antimigraine efficacy. However, due to its long half-life, on the long-term, frovatriptan showed a lower risk of recurrence than almotriptan.

## Appendix: list of study sites

Coordinator: Prof. B. Fierro (Palermo)

Investigators: M. Bartolini (Ancona), M.A. Giamberardino (Chieti), C. Lisotto (San Vito al Tagliamento), P. Martelletti (Roma), D. Moscato (Roma), B. Panascia (Catania) L. Savi (Torino), L.A. Pini (Torino), G. Sances (Pavia), P. Santoro (Monza), G. Zanchin (Padova), B. Fierro (Palermo).
